# Preparation of Carbon Aerogel Electrode for Electrosorption of Copper Ions in Aqueous Solution

**DOI:** 10.3390/ma12111864

**Published:** 2019-06-09

**Authors:** Ziling Cao, Chen Zhang, Zhuoxin Yang, Qing Qin, Zhihua Zhang, Xiaodong Wang, Jun Shen

**Affiliations:** Shanghai Key Laboratory of Special Artificial Microstructure Materials and Technology, Department of Physics Science and Engineering of Tongji University, Shanghai 200092, China; 1653583@tongji.edu.cn (Z.C.); chenzhang_tj@163.com (C.Z.); yangzhuoxin@tongji.edu.cn (Z.Y.); 1653574@tongji.edu.cn (Q.Q.)

**Keywords:** carbon aerogel, electrosorption, Cu^2+^ removal, capacitive deionization

## Abstract

Carbon aerogel (CA) has a rich porous structure, in which micropores and mesopores provide a huge specific surface area to form electric double layers. This property can be applied to the application of capacitive deionization (CDI). The adsorption effect of CA electrode on Cu^2+^ in an aqueous solution was explored for solving heavy metal water pollution. The CAs were synthesized by a sol-gel process using an atmospheric drying method. The structure of CAs was characterized by scanning in an electron microscope (SEM) and nitrogen adsorption/desorption techniques. The adsorption system was built using Cu^2+^ solution as the simulation of heavy metal pollution solution. The control variate method was used to investigate the effect of the anion species in copper solution, the molar ratio of resorcinol to catalyst (R/C) of CA, and the applied voltage and concentration of copper ion on the adsorption results.

## 1. Introduction

Water is the basis for the survival of all things, but nowadays, the per capita possession of freshwater resources in China is tiny, and these resources have been heavily polluted. Therefore, it is an urgent task to control sewage and achieve secondary utilization. At present, the main ion removal technologies include precipitation, electrolysis, membrane separation, and the ion exchange method [1]. Capacitive deionization (CDI) technology is an emerging desalination technology based on electrosorption. The main principle is that under the action of the electrostatic field, the charged ions in the solution move to the oppositely charged electrodes and enter the inside pores. They then form an electric double layer and are bound to the electrode to purify the solution [2]. Compared with traditional methods, CDI shows the advantages of high efficiency, no secondary pollution, and low cost. Nanoporous carbon aerogel (CA) is an ideal material for the CDI electrode [3,4]. Compared with other carbon materials, CA is highly porous and possesses high electrical conductivity, high electrolyte retention, high specific surface area, and a controllable pore size distribution. It is beneficial to form an electric double layer on the surface of the electrode that attracts a large amount of ions [5,6]. Therefore, it has received extensive attention in the field of ion adsorption purification [7,8,9]. The heavy metal ions in sewage, such as Cu^2+^, can also be removed by CDI [10,11,12,13]. In this paper, Cu^2+^ was used as the research object to study the application of CDI to remove heavy metal ions in an aqueous solution. CAs with different microstructures were synthesized by the sol-gel method using resorcinol and formaldehyde as a precursor. The prepared CA was used as a CDI electrode material to adsorb Cu^2+^ in aqueous solution, and its application in adsorbing heavy metal ions was explored.

## 2. Materials and Methods

### 2.1. Preparation of CA

The CA samples were synthesized by the previously reported technology [4,10]. Resorcinol (R) and formaldehyde (F) was used as a precursor in a molar ratio of 1:2, and a sodium carbonate solution, using as catalyst (C), was dissolved in deionized water, stirred for 2 h to synthesize resorcinol-formaldehyde (RF) into organic sol. The sol was injected into a glass mold, sealed, and placed in a drying oven at 30 °C, 50 °C and 90 °C for 24 h, 24 h and 72 h, respectively. The obtained RF organic wet gel was soaked with absolute ethanol for 3 d for solvent-exchange, and the ethanol solvent was renewed once a day. Then, the RF gel was dried at atmospheric pressure in a 50 °C oven for 3–5 days to get the RF organic aerogel. The CA was prepared by the carbonization of RF aerogel at 1000 °C for 3 h under N_2_ atmosphere protection. Then, it was activated at 1000 °C in a CO_2_ atmosphere for 2 h. The molar ratios of resorcinol to catalyst (R/C) in this paper were 300, 500, 800, 1000, and 1500. The mass fraction of R and F in the RF organic sol (M%) was 30. The obtained CA samples were labeled 330, 530, 830, 1030, 1530, respectively. 

### 2.2. Characterization

Morphology detection and pore structure analysis are significant characterization methods for nanoporous materials [14]. The morphology of CAs was characterized by a Plilips XL30 FEG scanning electron microscope (SEM, Royal Dutch Philips Electronics Ltd., Amsterdam, The Netherlands). The particle size and stack of different CAs were compared and analyzed from the SEM images. To more clearly reflect the sample characteristics, the CA samples were sprayed with gold for 80 s before testing. The specific surface area (SSA) of CAs were tested using a TriStar 3000 nitrogen adsorption analyzer (Micromeritics Instruments Corporation, Norcross, GA, USA) at 77 K and computed by Brunauer–Emmett–Teller (BET) algorithm, while the pore size distribution was calculated using the Barrett–Joyner–Halenda (BJH) method.

### 2.3. Electrochemical and CDI Adsorption Test

The CA block was crushed and ground into powder by a pulverizer (Yongkang Jiupin Industry and Trading Co. Ltd., Yongkang, China). The CA powders, polyvinylidene fluoride (PVDF) and acetylene black were mixed in a mass ratio of 8:1:1. An appropriate amount of 1-methyl-2-pyrrolidone was added and stirred for 12 h to prepare CA mixture slurry. The foamed nickel, the current collector for electrochemical tests, with a thickness of 1 mm was cut into rectangular shapes of 1 cm × 3 cm and then immersed in alcohol and acetone for 30 min, respectively, to remove impurities on the surface of the foamed nickel. Finally, they were washed with alcohol several times and dried in a cool place. The CA slurry was uniformly coated on the dried foamed nickel with an area of 1 cm × 1 cm. Then these coated foamed nickel sheets were placed in a vacuum oven at 100 °C for 10 h and pressed at a pressure of 15 MPa for 20 s to prepare CA electrodes for the electrochemical test. The electrochemical tests including cyclic voltammetry (CV) and electrochemical impedance spectroscopy (EIS) were performed with a standard three-electrode system, including a reference electrode (calomel electrode), a counter electrode (platinum electrode) and a working electrode (CA electrode). The electrolyte solution was KOH solution with a concentration of 6 mol/L. 

**Figure 1:**
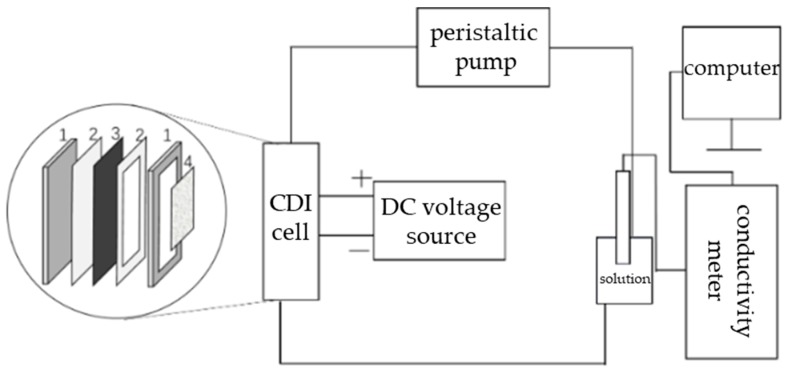
Capacitive deionization (CDI) system diagram and the framework of the CDI cell. Thereinto, 1 is a plexiglass plate as a support plate, 2 is a rubber gasket to prevent the water leakage, 3 is a carbon aerogel (CA) electrode as the working electrode, and 4 is a glass filament separator to prevent short circuiting.

The prepared CA slurry was uniformly coated on current collector graphite sheets (10 × 10 cm^2^) and dried in an oven at 100 °C under vacuum condition for 10 h to obtain CA electrodes for CDI experiments. Two same electrodes were mounted to a "sandwich" CDI cell in the order of plexiglass plate, CA electrode, plexiglass frame, glass filament separator, CA electrode, and plexiglass plate. The plexiglass frame with a central hollow kept a separation distance of 5 mm between the positive and negative electrodes. The rubber gaskets were clamped between the plexiglass and the electrode to prevent water leakage (as shown in Figure 1). CDI adsorption experiments were performed in a batch-mode circulatory system shown in Figure 1, which consisted of a CDI cell, solution tank, conductivity meter, peristaltic pump, and a direct current (DC) voltage source. The solution was flowed from the solution tank into the CDI cell and back to the solution tank, forming a circulation system. The solution flow rate was about 25 mL/min and constant for all the tests. The DC voltage source was used to provide DC voltage. The conductivity of the solution was detected in real time by the conductivity meter connected to the computer, and the data was recorded every minute. 

### 2.4. Preset of the Copper Solution

The concentration of the solution is linear with the conductivity in the low concentration range and could be converted by the concentration–conductivity calibration curve. The linear relationships between concentration and conductivity of CuSO_4_, Cu(NO_3_)_2_, and CuCl_2_ solutions, respectively, are as follows:

CuSO_4_:            Con. = 28.08392 + 0.60236c, r_1_ = 0.99631 (1)

Cu(NO_3_)_2_:          Con. = 17.80408 + 0.94524c, r_2_ = 0.99916 (2)

CuCl_2_:           Con. = 25.39195 + 1.34801c, r_3_ = 0.99957 (3)

where c represents the solution concentration (mg/L), Con. represents the conductivity of the solution (μS/cm), and r_1_, r_2_ and r_3_ are the correlation coefficients of the linear fitting.

## 3. Results and Discussion

### 3.1. Characteristics of CA

#### 3.1.1. Structural Characterization

**Figure 2:**
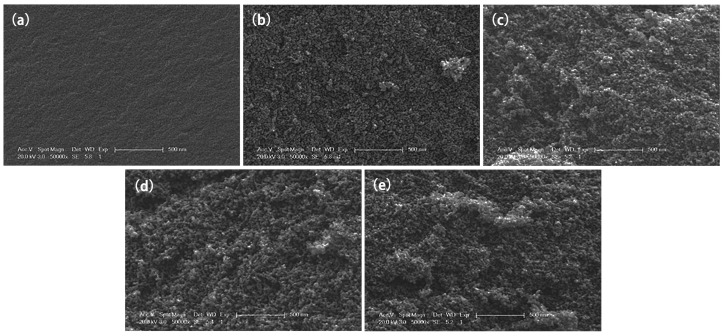
SEM image of different CAs. (**a**) 330; (**b**) 530; (**c**) 830; (**d**) 1030; (**e**) 1530.

The surface morphology of the electrode material is an important factor affecting the electrosorption capacity. Figure 2 is the SEM image of 330, 530, 830, 1030 and 1530. It can be seen from the figure that CA has a three-dimensional network-like pore structure that is crosslinked by many small particles. The microstructure of CA is regulated and controlled by R/C. As the R/C increases, the carbon particles grow, the pores between particles get larger, and thus, the structures of CAs become looser. When the M% is fixed, the larger the R/C, the smaller the amount of catalyst. As a result, R and F in the precursor solution can be sufficiently reacted to form larger particles. Therefore, the pores which are stacked by cross-linking carbon particles are larger, resulting in a looser structure. Table 1 shows the SSA and pore structure parameters of 330, 530, 830, 1030, and 1530. With the increase of R/C, the micropore SSA firstly improves and then falls off. The 1030 sample has the largest pore volume (3.41 cm^3^/g), micropore SSA (1083 m^2^/g), and volume (0.55 cm^3^/g), and average pore diameter, which provides the fast pathway for ion transfer that facilitates electrosorption.

#### 3.1.2. Electrochemical Tests

**Figure 3:**
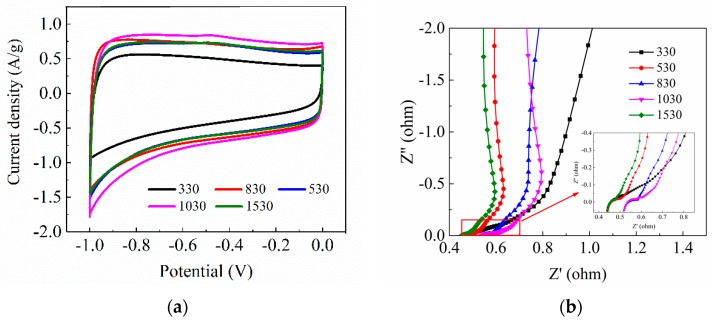
(**a**) The cyclic voltammetry (CV) curves of CA samples with different R/C in 6 mol/L KOH solution at the scan rate of 10 mV/s and (**b**) the EIS Nyquist plots of CAs with different R/C.

Figure 3a displays the CV curves of CA samples with different R/C in 6 mol/L KOH solution at the scan rate of 10 mV/s. It can be found that almost all the curves show symmetrical rectangular shape, indicating that the CA electrodes have excellent electric double layer performance. There are no obvious redox peaks in the five curves, which manifests that no redox reaction and faradaic pseudo-capacitance occur. It is further demonstrated that ions are trapped in the electrode by forming an electric double layer during electrosorption. The specific capacitance SC of each CA electrode can be calculated as follows:

(4) SC=∫IdV/(m·v)

where *I* is the current (A), *V* is the voltage (V), m is the mass of CA in the electrode material (g), and v is the scan rate (V/s). The specific capacitance of CA samples with different R/C is shown in Table 2. The specific capacitance tends to increase first and then decrease with the increase of R/C. The specific capacity of the 1530 sample is significantly reduced. The 1030 sample has the largest specific capacitance, attaining 156 F/g (at the scan rate of 10 mV/s), indicating that 1030 has the best electrosorption performance. The variation trend of specific capacitance for CA electrodes is consistent with the changes in the amount of micropores (Table 1), which proves that the electrosorption performance has a great relationship with the pore volume and micropore amount of aerogel.

Figure 3b shows the EIS Nyquist plots of CAs with different R/C. The semi-circular arc at high frequency represents the charge transfer resistance at the electrode/solution interface. All CA electrodes show very small diameters of the semicircles, suggesting the electrodes have a really small charge transfer resistance. The straight line with a slope of about 45° in the middle frequency region implies the ion diffusion resistance in the electrode, and the vertical straight line at low frequency reflects the electric double layer capacitance characteristics. The five samples in the figure all show satisfactory shape, indicating that these CA electrodes exhibit good electrical conductivity and excellent electrochemical performance [15,16]. 

### 3.2. Adsorption Results of Cu^2+^

#### 3.2.1. Effect of Anions on Adsorption Capacity

**Figure 4:**
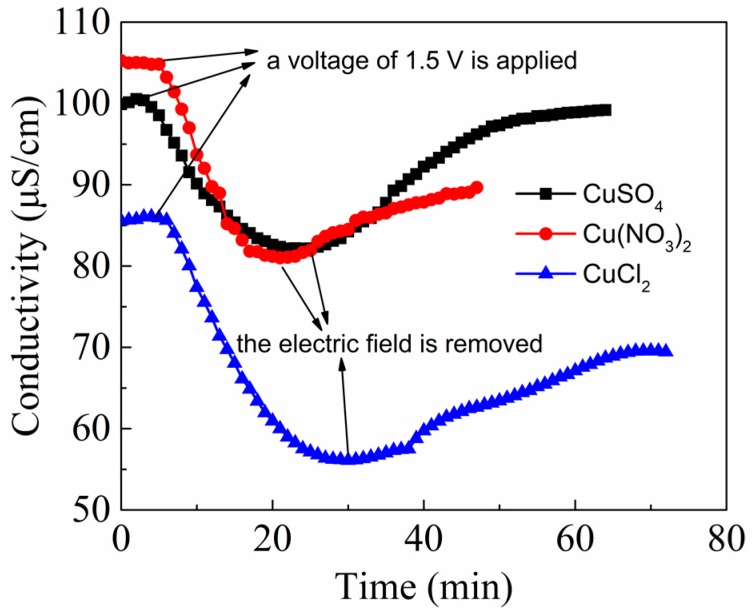
The adsorption curves of CA electrodes for three copper solution.

**Figure 5:**
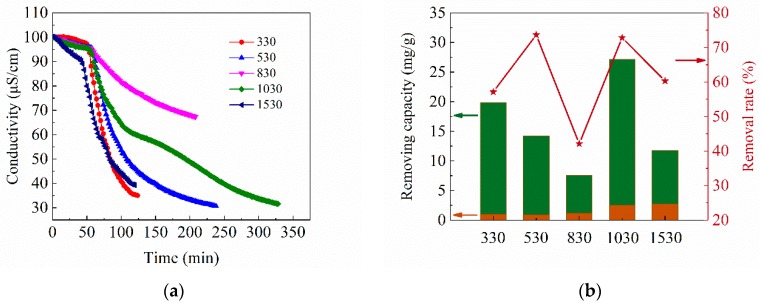
(**a**) Adsorption curves and (**b**) the SAC and removal rate of CAs with different R/C in CuSO_4_ solution. The orange area in (**b**) represents the specific adsorption during the open circuit, and the green area represents the electrosorption capacity.

In order to study the effect of anion species on the adsorption capacity of CA, CuSO_4_, CuCl_2_ and Cu(NO_3_)_2_ solutions were used for electrosorption experiments. The CA electrode used in this part was the 1530 sample. The volume of copper solution was 100 mL, and the concentration was 100 mg/L. Figure 4 shows the electrosorption process of the CA electrode in the copper solutions with different anion species. When a voltage of 1.5 V is applied, ions in the solution are rapidly adsorbed to the surface of electrodes, and the conductivity of the solution decreases. After some time, the conductivity of the solution reaches equilibrium, and then the electric field is removed. The ions diffuse freely from the electrodes to the solution due to the loss of electrostatic force, and the conductivity increases. Table 3 lists the salt adsorption capacity (SAC) of CA for Cu^2+^ in the three solutions. The SAC mentioned here is defined as the total mass of ions during the electrosorption, that is, the mass of ions adsorbed per gram of CA. As can be seen from Figure 5 and Table 3, the ion adsorption rate in the solution with SO_4_^2−^ is faster than that in the solution with NO_3_^−^ and Cl^−^. It is because the valence state of SO_4_^2−^ is higher than the other two. As a result, the driving force on ions in the solution with SO_4_^2−^ applied by the external electric field is stronger, the rate of ions moving toward the electrodes is faster, and the adsorption rate is also higher. The SAC gets sequentially weakened in the solution with SO_4_^2−^, NO_3_^−^ and Cl^−^. In addition, the CDI electrosorption is impacted by the hydrated ionic radius (HIR) and ion mass. The HIR of NO_3_^−^ and Cl^−^ (335 pm and 332 pm, respectively) differs slightly, while the ion mass of NO_3_^−^ (62.004 amu) is greater than that of Cl^-^ (35.453 amu). Thus, the adsorption amount of ions by the CA electrode in Cu(NO_3_)_2_ solution is slightly larger than that in the CuCl_2_ solution. Therefore, in the following experiments, the CuSO_4_ solution is selected as the adsorption solution.

#### 3.2.2. Effect of CA Structure on Adsorption Capacity

The CA electrodes with different R/C were used for CDI electrosorption experiments in CuSO_4_ solution with the concentration of 100 mg/L. The applied voltage was 1.2 V, and all samples were subjected to specific adsorption for 1 h before charging. The adsorption curves are shown in Figure 5a. All curves show a similar trend: the conductivity of the solution decreases slowly at first, while drops rapidly after applying voltage, and finally, do not fall anymore to achieve a minimum. The specific adsorption during an open circuit is due to the reaction of the functional groups on the CA surface with the copper ions. After applying the voltage, the electrosorption occurs and dominates on CA electrodes due to the presence of the electric field force. The SAC of each CA sample is displayed in Figure 5b. The 1030 sample has the highest SAC, up to 29.7 mg/g. Noked pointed out that micropores were mainly used to store charge during CDI electrosorption [17]. The 1030 sample has the largest amount of micropores, leading to the largest SAC. The 530 sample has the highest salt removal rate η (defined as the ratio of the reduction in solution concentration to the initial concentration), probably due to the large amount of CA on the electrode. In this experiment, the maximum removal rate of a single CDI cell can reach 73.6%. It can be predicted that when multiple CDI cells are connected in series, the removal rate of CA to Cu^2+^ will achieve a very desirable result.

#### 3.2.3. Effect of Applied Voltage and Cu^2+^ Concentration on Adsorption Capacity

**Figure 6:**
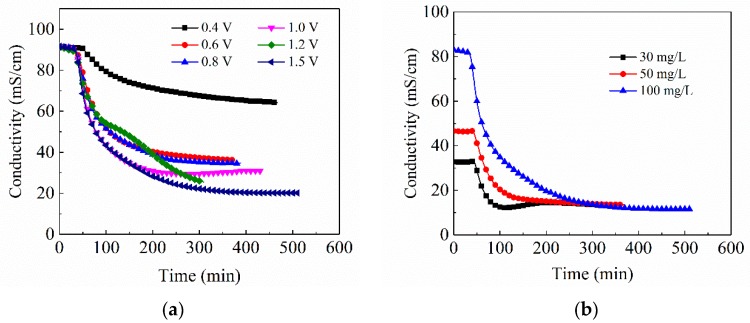
Adsorption curves of different CAs (**a**) for CuSO_4_ solution and (**b**) the adsorption curves of carbon aerogel electrode for CuSO_4_ solution at different voltages.

Operating conditions have a major impact on CDI performance. Therefore, the electrosorption capacity of CA in CuSO_4_ solution with the concentration of 100 mg/L under different applied voltage was investigated with the 1030 sample. The adsorption curves under the voltage of 0.4–1.5 V are displayed in Figure 6a. Since the CA samples are the same, the specific adsorption capacities in the front part of the curves are similar. Under different voltages, the adsorption rate is accelerated, with the increasing voltage due to the intensive electric field force. Combined with the SAC calculation at different voltages in Table 4, the electrosorption capacities of CA climb up and then decline as the applied voltage increases. CA electrode achieves the largest SAC under the applied voltage of 1.2 V. The SAC decreases at 1.5 V, which may be ascribed to the electrolytic reaction of water caused by the too large voltage. The applied voltage is an important factor affecting the adsorption capacity, adsorption rate, and removal rate of the CA electrode. Therefore, for different CDI systems, the corresponding optimal operating voltage should be ascertained.

In addition to the applied voltage, the concentration of Cu^2+^ in the solution also affects the CDI adsorption performance. Figure 6b shows the adsorption curves of the same CA in CuSO_4_ solution with different concentrations of 30 mg/L, 50 mg/L and 100 mg/L at the applied voltage of 1.2 V. It can be found that the adsorption rate of copper ions gradually increases with the increase of solution concentration. As the concentration gets larger, the capacity of specific adsorption and total adsorption, and removal rate gradually increases. When the applied voltage is fixed, the move rate of ions toward the electrode surface is similar. The higher the concentration of the solution, the more the number of ions moving toward the electrode per unit time, so the higher the adsorption rate and the adsorption capacity.

## 4. Conclusions

The CAs with different R/C were prepared by a sol-gel method. The structure of CA was characterized by SEM, and the control effect of R/C on the structure of CA was discussed. CA is composed of cross-linking nanoparticles, and its pore structure consists of micropores inside nanoparticles, mesopores, and macropores between the particles. The R/C in the precursor solution affects the formation of particles and cross-linking between particles, which further impacts the structure and properties of CAs.

CV tests show that the prepared CA samples had good electrochemical properties. The 1030 sample has the highest specific capacity of 156 F/g at the scanning rate of 10 mV/s. 

Through the CDI electrosorption experiments of the copper solution, the effects of anion species, the microstructure of CAs and operating conditions (including applied voltage and Cu^2+^ concentration) on the electrosorption capacity were explored. Moreover, the optimal parameter ranges for the corresponding conditions for the best adsorption capacity and removal amount were obtained. Among different anions, the order of adsorption effect is SO_4_^2−^ > NO^3−^ > Cl^−^. In different CAs, the CA electrode with R/C of 1000 has the best adsorption capacity. For different operating conditions, the best adsorption performance is obtained at the applied voltage of 1.2 V under the solution concentration of 100 mg/L. The CDI adsorption experiments of copper ions by CAs in this work show that CAs have great development potential in the field of heavy metal ion treatment.

## Figures and Tables

**Table 1:** Specific surface area and pore structure parameters of different CAs.

CAs	S_BET_^1^ (m^2^/g)	S_mic_ (m^2^/g)	V_t_ (cm^3^/g)	V_mic_ (cm^3^/g)	D_ave_ (nm)
330	832	416	0.36	0.21	3.5
530	2177	819	3.0	0.41	8.6
830	1496	928	1.77	0.46	11.3
1030	2057	1083	3.41	0.55	13.3
1530	2188	920	2.87	0.45	9.4

^1^ S_BET_: SSA calculated by multi-point BET method; S_mic_, V_mic_: Micropore SSA and volume obtained using t-plot calculation; V_t_: Total pore volume; D_ave_: Average pore diameter.

**Table 2:** The specific capacitance of different CAs.

Sample (R/C)	Specific Capacitance/(F·g^−1^)		
	10 mV·s^−1^	50 mV·s^−1^	100 mV·s^−1^
330	93	75	65
530	132	115	106
830	141	128	118
1030	156	135	123
1530	133	115	103

**Table 3:** The salt adsorption capacity (SAC) of CA electrodes for different copper solution.

Anion Species	SO_4_^2−^	NO_3_^−^	Cl^−^
Concentration (mg/L)	100	100	100
Total SAC at 1.2 V (mg/g)	7.24	5.50	5.27

**Table 4:** The SAC and removal rate of CA electrodes for CuSO_4_ solution at different voltages.

Voltage (V)	SAC (mg/g)	η (%)
0.4	15.97	31.7
0.6	17.36	64.3
0.8	22.99	61.5
1.0	17.86	72.6
1.2	29.70	72.8
1.5	25.78	85.4
